# Use of a *Pleurotus ostreatus* Complex Cell Wall Extract as Elicitor of Plant Defenses: From Greenhouse to Field Trial

**DOI:** 10.3390/molecules25051094

**Published:** 2020-02-29

**Authors:** Céline Faugeron-Girard, Vincent Gloaguen, Rromir Koçi, Julien Célérier, Anaïs Raynaud, Charlotte Moine

**Affiliations:** 1 Université de Limoges, Laboratoire Peirene (EA7500), Faculté des Sciences et Techniques, 123, avenue Albert Thomas, 87060 Limoges CEDEX, France; vincent.gloaguen@unilim.fr (V.G.); rromir.koci@unilim.fr (R.K.); 2 COVERTIS SAS, Technopole d’ESTER, 1 avenue d’Ester, 87069 Limoges CEDEX, France; julien.celerier@covertis-lab.fr (J.C.); anais.raynaud@covertis-lab.fr (A.R.); charlotte.moine@covertis-lab.fr (C.M.)

**Keywords:** *Pleurotus ostreatus*, glucan-rich complex, plant defenses

## Abstract

Fungi constitute an abundant source of natural polysaccharides, some of them harboring original structures which can induce responses in mammalian or plant cells. An alkaline extract from the edible mushroom *Pleurotus ostreatus* has been obtained and called Pleuran complex cell wall extract (CCWE). It consists of a glucan-peptide complex whose components fall in a quite broad range of molecular weights, from 30 to 80 kDa. Pleuran extract has been tested on cultivated plants in laboratory conditions and also during field trial for its capacity to stimulate plant defenses in response to pathogen attack. Following Pleuran CCWE treatment, enhanced levels of various biochemical markers associated with plant responses have been observed, including enzymatic activities (e.g., peroxidase) or expression of some pathogenesis-related genes. In addition, during field experiments, we have noticed significant reductions in disease symptom levels in relation to different plant/pathogen systems (wheat/septoria, vine/mildew). These results confirmed that Pleuran CCWE could be used as an elicitor of plant defenses and could help in reducing pesticide applications against plant pathogens.

## 1. Introduction

Edible mushrooms are widely consumed all over the world and sometimes are cultivated in large amounts. An edible mushroom constitutes the aerial part of a fungus which carries the spore-bearing fruiting body. An important part of the biomass of these mushrooms is the fungal cell wall which consists of a complex assembly of different polysaccharides (α- and β-glucan, galactomannan and chitin) and various proteins [1,2,3]. In mushrooms, β-glucans are composed of a linear β1→3 glucan backbone harboring β1-6 glucan side chains. Linear (1→3)α-D-glucans have also been described [3]. Chitin is composed of long chains of β(1→4) linked N-acetylglucosamine units. All of these polysaccharides are closely associated with fungal cell walls [4].

Some of these polysaccharides have been proposed for medicinal applications, owing to their hypocholesterolemic or immunomodulatory effects, and their antibacterial properties [5,6,7,8]. These polysaccharides could also serve as attractive substitutes for the environment- and health-unfriendly phytochemicals which are currently widely used for crop protection. Chitin, β-glucan or their derivatives can be recognized by a plant cell as pathogen signals, and can elicit plant defenses [4,9]. Designated as microbial-associated molecular patterns (MAMPs), they interact with the so-called plant-recognition receptors (PRR) [10,11]. Then, rapid intracellular responses are triggered, notably variation in cytosolic Ca^2+^ concentration, production of reactive oxygen species, and activation of MAPK (mitogen-activated protein kinases). These early events are followed by an enhanced synthesis of pathogenesis-related proteins (PR), some of them, e.g., glucanase or chitinase, directly involved in defense against pathogens, are able to degrade components of fungal cell walls. Recognition of MAMPS induces the additional synthesis of phytoalexins, secondary metabolites endowed with antimicrobial properties. The plant cell wall is also reinforced by lignification which limits its degradation by the pathogen. These plant defense responses at the site of pathogen attack also imply the production, release and transport of plant hormonal signals such as salicylic acid to the distal parts of the plant through a process called systemic acquired resistance (SAR) [12]. SAR is known to be a long-lasting and broad-spectrum resistance; this characteristic is of great advantage since these signal molecules could also be used for plant protection purposes.

MAMPs have been recently used to enhance the resistance of cultivated plants in a process called MAMP-triggered Immunity (MTI). This strategy helps the plant to defend itself from pathogen attack and diminishes fungicide application [13]. Some of these MAMPs have already been authorized for agricultural purpose. This is the case for laminarin, a β-glucan extracted from brown algae [14,15], or COS-OGA which associates oligogalacturonates (OGA), i.e., fragments of pectins, with partially acetylated chitosan (COS), derived from the exoskeletons of crustaceans whose structure mimics the fungal cell wall [16,17]. In this case, COS, a MAMP, is associated with OGA, a DAMP (damage-associated molecular pattern), also recognized as a signal of pathogen attack which enhances plant defenses.

The aim of this study was to obtain a β-glucan-enriched soluble extract from the edible mushroom *Pleurotus ostreatus*, and to determine its ability to stimulate plant defenses in response to pathogen attacks. The biological activity of the extract obtained was observed in controlled conditions on plantlets grown in a greenhouse and also during field trials.

## 2. Results and Discussion

### 2.1. Yields and Process Scale-Up

The main parameters of extraction process realized at laboratory and pilot scales are summarized in Table 1. Each one of these extracts, obtained by alkaline extraction of *Pleurotus ostreatus* has been called Pleuran complex cell wall extract (CCWE).

First of all, extraction process was realized successfully at pilot scale with a size increasing factor of more than 10. Indeed, the process was scaled-up from 0.2 kg (laboratory) to 2.8 kg (pilot) dried *Pleurotus*, paying attention to the ratio volume produced to raw material, that we kept quite constant (8.5 and 8.0 L/kg, respectively), while an increase in dry residue concentration of the liquid extract, from 32.9 to 40.7 g/L, was achieved.

### 2.2. β-Glucan and Crude Protein Contents

Contents of glucans and crude proteins of laboratory and pilot Pleuran CCWE, are reported in Table 2. For both extracts, the proportions of α-glucans was low compared with β-glucans. These compounds represent a significant part of the whole extract. Chemical characterizations of the two extracts gave quite similar results. Major glucans described in *Pleurotus ostreatus* are branched (1–3), (1-6)-β-D-glucans [18,19]. They consist of a backbone of β(1-3)-linked D-glucopyranose units, in which O-6 is substituted with a single D-glucopyranosyl group every fourth residue [20]. Other different polysaccharidic structures from *Pleurotus* have been observed, for example a linear α-(1-3)-linked D-glucan, isolated from P. *ostreatus* and P. *eryngii* [21], and a β-(1-3),(1-6)-linked glucan, extracted from *P. ostreatus* [19]. Crude protein content of Pleuran CCWE represents 23% of the whole extract. Nevertheless, this fraction is actually composed of a complex mixture of proteins, peptides and free amino acids. Overall, the sum of glucans and crude proteins represents at least 40% of the dry mass, the rest is constituted by other components such as mono-, polysaccharides and minerals.

### 2.3. Monosaccharide Composition

Analyses of monosaccharide composition are reported in Table 3. For both processes (laboratory and pilot extracts), glucose was the main monosaccharide found in Pleuran CCWE (>90%). Smaller amounts of mannose and galactose were also detected. This was consistent with the fact that polysaccharides of Pleuran CCWE are mainly glucans with the presence of a minor fraction of heteropolysaccharides, mainly mannogalactans [3].

### 2.4. Steric Exclusion Chromatography/Multi-Angle Light-Scattering/Differential Refractive Index (SEC/MALS/DRI) Analysis

**Figure 1:**
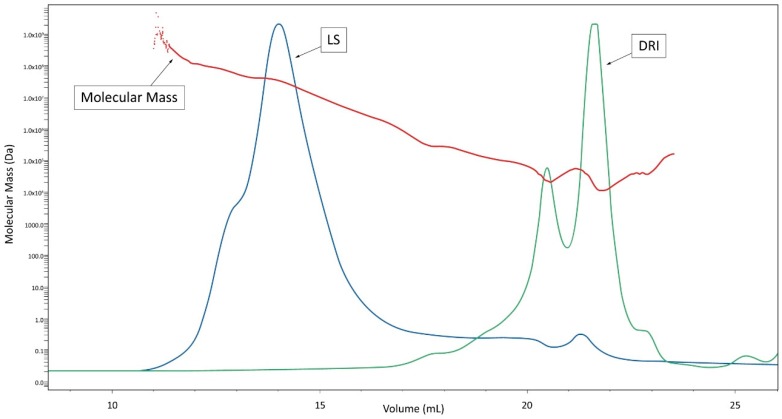
Steric exclusion chromatography/multi-angle light scattering/differential refractive index (SEC/MALS/DRI) analysis of Pleuran CCWE (DRI = differential refractive index, LS = light-scattering signal). The profiles shown are representative of two independently obtained extracts.

The results obtained by steric exclusion chromatography (SEC) coupled with differential refractive index (DRI) and light-scattering (LS) detections are shown in Figure 1. DRI chromatogram showed 3 distinct fractions: F1 between 11 and 17 mL, F2 between 17 and 21 mL and F3 comprised between 21 and 23.5 mL. Multi-angle light scattering (MALS) analyses of the main fractions F2 and F3 were consistent with average molecular masses of 80 and 30 kDa, respectively. These fractions were characterized by a low light scattering. At lower elution volumes (Ve), between 11 and 17 mL (fraction F1), we have recorded a flat DRI signal associated with an important LS peak. The combination of these two signals indicated that this fraction contained low concentrations of very large compounds. This LS signal (at 14 mL) was linked with a slight re-increase of molecular mass. Also, a weak LS signal between 21 and 22 mL (F3) showed a slight increase in molecular mass. These observations are inconsistent with the steric exclusion chromatography of polydisperse homopolymers, for which a continuous decrease in molecular mass is concomitant with increasing elution volume. This could rather indicate the presence, in F3, of compounds, with higher Ve and then smaller Vh (hydrodynamic volume), which possess molecular masses higher than those of compounds found in F2. This conclusion implies that the relatively small size of these molecules is the consequence of their compact conformation, which is consistent with the known packed structure of protein-polysaccharide aggregates.

### 2.5. High-Performance Liquid Chromatography (HPLC)-SEC Analysis

**Figure 2:**
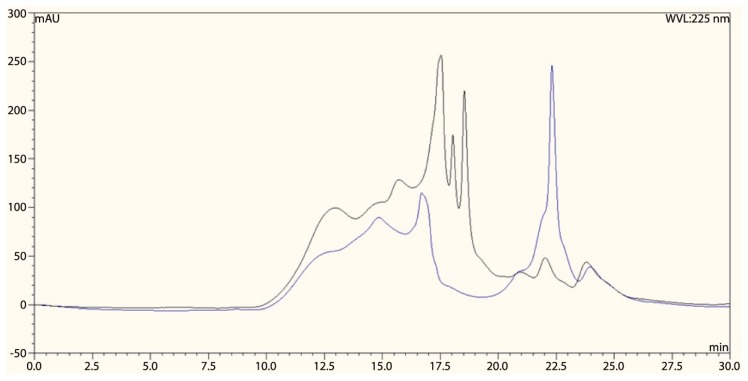
Chromatogram of Pleuran CCWE analysis by high-performance liquid chromatography (HPLC)-SEC (ultraviolet (UV) detection λ = 225 nm); Black: native Pleuran CCWE; blue: Pleuran CCWE after NaOH treatment. The profiles shown are representative of two independently obtained extracts.

Analysis of Pleuran CCWE by steric exclusion chromatography, coupled with ultraviolet (UV) detection (λ = 225 nm), is shown in Figure 2. Native Pleuran CCWE chromatogram (black curve) displayed a complex pattern in the 10–20 min area and, in particular, several intense peaks were recorded between 17 and 20 min; after 20 min, a few small peaks were also observed. After incubation in 0.2 M NaOH, the chromatogram of Pleuran CCWE (blue curve) showed a decrease in intensity of the the peaks between 10 and 20 min, along with the vanishing of the strong peaks between 17 and 20 min, and the appearance of a large peak at 22 min. This observation could indicate that large glucan-peptide aggregates present in native Pleuran CCWE undergo dissociation under alkaline treatment and give rise to smaller species, as illustrated by the appearance of the large peak at 22 min (blue curve). These conclusions are consistent with the results obtained by SEC/MALS/DRI analysis. Such glucan-peptide complexes have already been described in aqueous extracts of *Pleurotus* species [21].

### 2.6. Biological Activities of Pleuran Complex Cell Wall Extract (CCWE)

#### 2.6.1. Controlled Conditions

**Figure 3:**
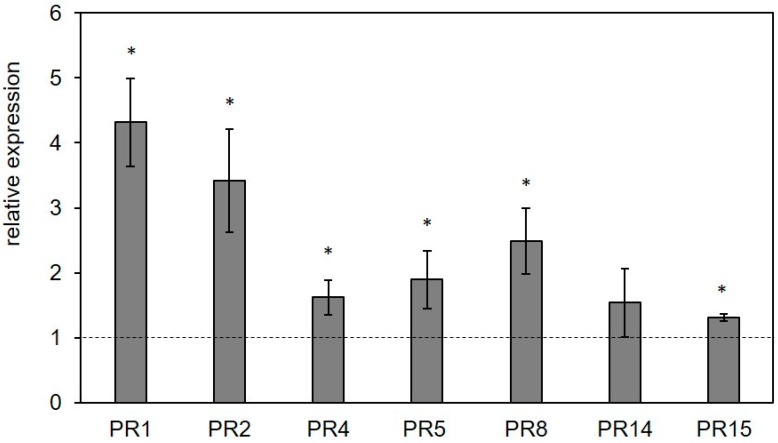
Relative expression levels of some pathogenesis-related (PR) genes in tomato leaves subjected to Pleuran complex cell wall extract (Pleuran CCWE) treatment (foliar application) compared to control (treatment with water; dotted line). All the plants were inoculated by infiltration of *Botrytis cinerea* conidia 24 h before analysis. Bars represent the average +/− standard deviation (*n* = 3). * means significative difference with control (*p* < 0.05).

The biological activity of Pleuran CCWE was firstly examined in controlled conditions on tomato plants cultivated in a greenhouse. Its capacity to elicit the plant defense responses was determined by spraying the Pleuran CCWE solution (350 mg·L^−1^) on tomato leaves (three times at two days intervals) before inoculation of plants with *Botrytis cinerea* (three days after the last spraying). One day later, a few leaves (different from the inoculated ones) were collected to determine by quantitative polymerase chain reaction (qPCR) the expression level of the pathogenesis-related genes (PR) *PR1, PR2, PR4, PR5, PR8, PR14* and *PR15*. Expression of all these PR genes was significantly stimulated, except PR14 whose expression did not differ from the control (Figure 3). To determine the intrinsic capacity of Pleuran CCWE to stimulate plant defenses in the context of a pathogen attack, different concentrations of Pleuran CCWE were applied on tomato plants. The reference dose, called 1N corresponding to 350 mg·L^−1^ expressed in dry matter, was compared with two solutions containing 700 mg·L^−1^ (2N) or 175 mg·L^−1^ (0.5N). Control plants were sprayed with a blank formulation (BF) consisting of an aqueous mixture containing only the surfactant and the preservative used to prepare the Pleuran CCWE solution.

Peroxidase activity has been chosen as a biomarker of plant defenses as it is often cited as an enzyme whose activity could be enhanced under biotic stress conditions [22]. Peroxidase can be implicated in the reinforcement of the plant cell walls by cross-linking cell wall components, in the synthesis of anti-microbial molecules such as phytoalexins and also in the metabolism of reactive oxygen species that could limit pathogen development at the infection site [22].

**Figure 4:**
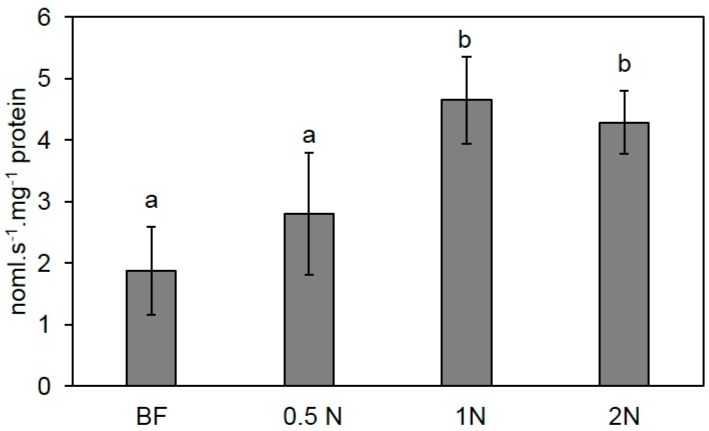
Peroxidase specific activities expressed in nmol.s^−1^.mg^−1^ protein in tomato leaves treated with Pleuran complex cell wall extract before inoculation with *Botrytis cinerea*. The leaves were collected seven days after inoculation. BF: blank formulation; 1N corresponds to a concentration of 350 mg.L^−1^ of Pleuran CCWE in the sprayed solution. Data are means of 6 replicates, +/− standard deviation. Different letters indicate significant differences at *p* < 0.05.

Pleuran CCWE treatment at dose 1N triggered an increase of peroxidase activity which was significantly different from the control, BF (Figure 4). But twice this concentration (2N) did not lead to increased activity. However, halving the concentration significantly reduced the peroxidase activity in tomato leaves. Therefore, the dose N (corresponding to 350 mg·L^−1^ of Pleuran CCWE) was finally retained for further experiments.

**Figure 5:**
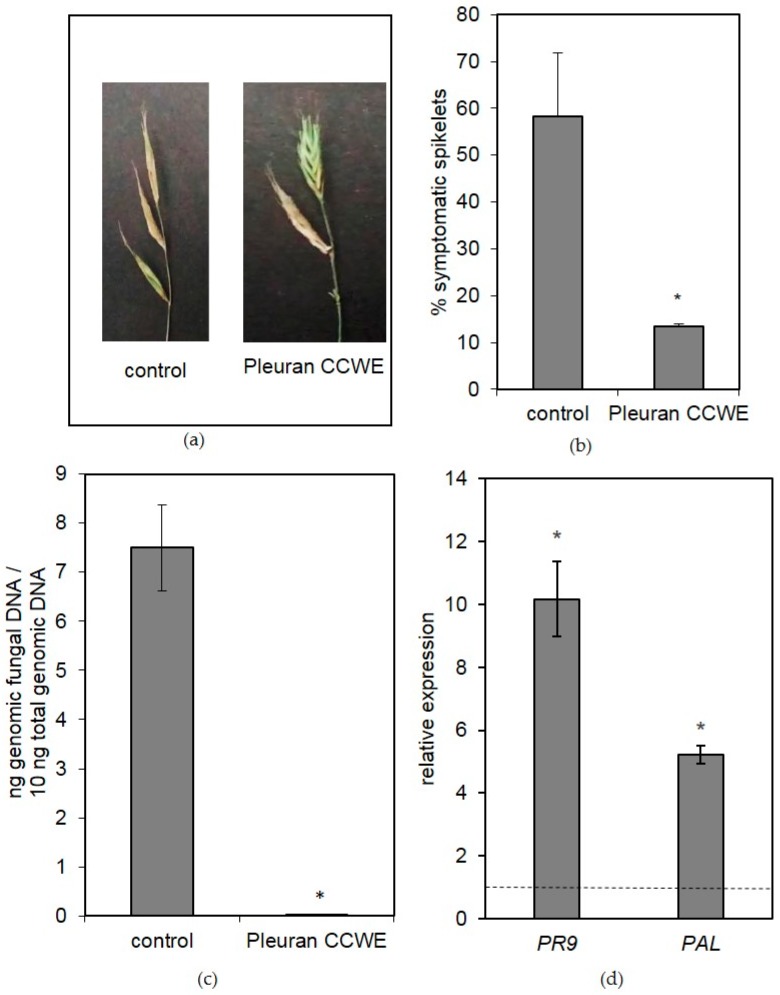
*B. distachyon* responses to a Pleuran complex cell wall extract (CCWE) pre-treatment before inoculation with *Fusarium graminearum.* (**a**) and (**b**): symptoms observed on spikelets (10 days post-inoculation) (**c**) Quantification of fungal biomass in plants determined by quantitative polymerase chain reaction (PCR) (**d**) Relative expression levels of *Phenylalanine-Ammonia-Lyase* (*PAL*) and *peroxidase* (*PR9*) genes in *B. distachyon* leaves 48 h post-inoculation with *Fusarium graminearum* for plants pre-treated with Pleuran CCWE related to the control (not treated; dotted line). Each value is the mean of two replicates (ten plants per replicate) and vertical bars are standard deviation. * means significant difference with control (*p* < 0.05).

Another plant model has been subjected to Pleuran CCWE treatment in controlled conditions, *Brachypodium distachyon,* which was chosen as a model of monocotyledon species. In the context of an attack by a pathogen such as *Fusarium graminearum*, a pre-treatment by Pleuran CCWE is efficient in reducing the symptoms on spikelets (Figure 5a,b). The development of the fungus is considerably reduced in plants pre-treated with Pleuran CCWE compared with the control (Figure 5c). This could be explained by a stimulation of the expression of some PR protein genes such as *PR9* coding for a peroxidase or *PAL* coding for phenylalanine-ammonia lyase (Figure 5d). The latter protein catalyzes the first reaction of the phenylpropanoid pathway i.e., the conversion of phenylalanine to cinnamic acid which, in turn, leads to the synthesis of phytoalexins (with antibacterial activities) or to the synthesis of monolignols involved in the formation of lignin and thought to reinforce the plant cell wall [23].

#### 2.6.2. Field Trials

**Figure 6:**
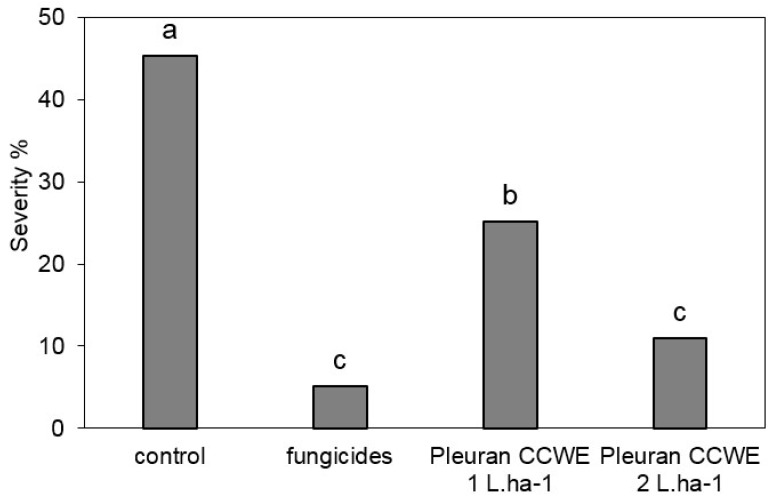
Severity of attacks by Septoria (*Septoria tritici*) on wheat in percentage on leaf area (T1) (field trial, notation 16 June 2017). Treatments were applied on 9 and 30 March and on 29 April 2017: control (no treatment), fungicides (Cherokee 2 L·ha^−1^ + Adexar 2 L·ha^−1^), Pleuran complex cell wall extract (CCWE) at concentrations either 1 L·ha^−1^ or 2 L·ha^−1^. Means followed by same letter or symbol do not significantly differ (*p* = 0.05, Student–Newman–Keuls).

As Pleuran CCWE was efficient to stimulate the plant defenses in the context of an attack by a pathogen in controlled conditions, a scale-up strategy was rolled out to confirm this activity at open field scale. Wheat (*Triticum aestivum* L.) was examined as this plant constitutes a worldwide-cultivated cereal crop. During this field trial (in 2017), the cold weather conditions led to a medium disease pressure: 45.3% of severity was observed for the control on 16 June (Figure 6). Pleuran CCWE had previously been applied by foliar spraying with two different doses levels: 1 L·ha^−1^ or 2 L·ha^−1^ (corresponding to doses of 35 and 70 g·ha^−1^, respectively). Application was repeated three times (on 9 March and 30 March and then on 29 April). A positive control was also devised by spraying wheat with two conventional fungicides, namely Cherokee^®^ (on 30 March) and Adexar^®^ (on 29 April). Figure 6 shows the evaluation of Septoria symptoms observed on wheat leaves on 16 June. As expected, fungicide treatments were efficient enough to reduce the severity of symptoms to 5.1%, corresponding to 89% efficacy. The efficacy of Pleuran CCWE treatment with the dose 2 L·ha^−1^ was 76%, equivalent to fungicide treatment, as the severity of Septoria symptoms in these conditions was statistically the same as the one obtained after fungicide application, although slightly higher (10.9% of severity). Also, the efficacy of Pleuran CCWE seemed to be proportional to the dose applied on wheat plants since the dose 1 L·ha^−1^ led to a lesser efficacy (45%) (Corresponding to 25.1% severity) but nevertheless significant compared with the control.

**Figure 7:**
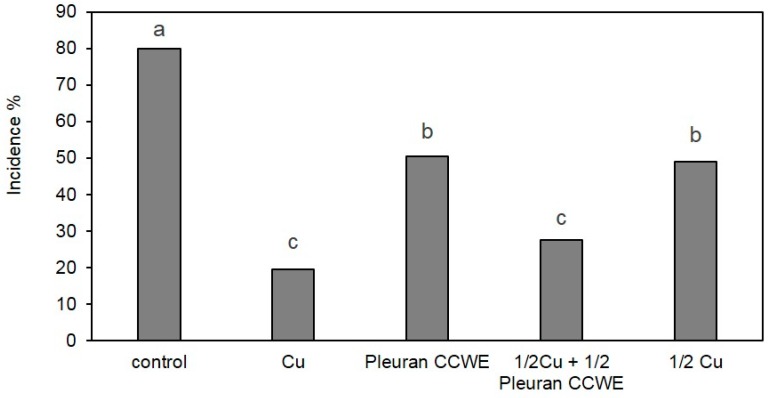
Effect of Pleuran CCWE treatment on incidence of downy mildew on grapevine (field trial, notation 20 July 2015) compared with conventional treatments based on Cu and alternative treatment combining Cu and Pleuran CCWE. % of attacked grapes. (Newman–Keuls test).

Vine (*Vitis vinifera*) is one of the most important fruit crop in the world. Its response to Pleuran CCWE treatment has also been evaluated during a field trial realized in 2015 in the south of France; more precisely, we assessed the mildew symptoms caused by the pathogen *Plasmopara viticola*. During the year 2015, symptoms on untreated plants (control) were sufficient to evaluate the efficacy of the treatments. For example, on 20 July, the incidence of mildew attacks on grapes in control conditions was estimated at 80% and their severity to 18.6% (Figure 7). Preventive treatments were applied to a few other vine spots: foliar spraying of Pleuran CCWE at dose N (corresponding to 350 mg.L^−1^ in the sprayed solution) realized every 8 to 10 days initiated by mid-May led to a significant reduction in incidence of mildew attacks on grapevine (50.5% for pleuran CCWE compared to 80% for control). The conventional treatment based on CuSO_4_ was more efficient (only 19.5% of incidence in this case) but this treatment led to reaching the authorized upper limit for this conventional fungicide on vine. In order to reduce fungicide use, an alternative treatment was also applied during this field trial: it consisted in three applications of Cu and three applications of Pleuran CCWE beginning by mid-May (instead of 6 applications of Cu in conventional conditions). The reduction of the incidence of mildew symptoms was statistically equivalent to the Cu treatment, despite the fact that incidence was a little higher (27.5% for Pleuran CCWE + Cu compared to 19.5% for Cu alone). The efficiency of only three applications of Cu (1/2 Cu, Figure 7) was estimated for comparison purposes: as expected, the intensity of symptoms was reduced, compared with the control and a similar level was obtained with Pleuran CCWE treatment alone (49% incidence). This experiment is a clear demonstration that Pleuran CCWE could be proposed as a complementary treatment to reduce fungicide application while maintaining the development of the pathogen sufficiently low to be used for agricultural purpose with a slight impact on crops. This strategy could also be of significant help in reducing phytochemical residues in the environment because Cu, due to its wide use for vine protection, constitutes a pollutant which accumulates in the soils of vineyards [24].

## 3. Materials and Methods

### 3.1. Mushroom Raw Material

Mushrooms (*Pleurotus ostreatus* var.) were obtained from Champicreuse (Saint-Yrieix-la-Montagne, France). Immediately after collection, mushrooms were dried at 30 °C in ventilated area.

### 3.2. Extraction of Pleuran CCWE

**Figure 8:**
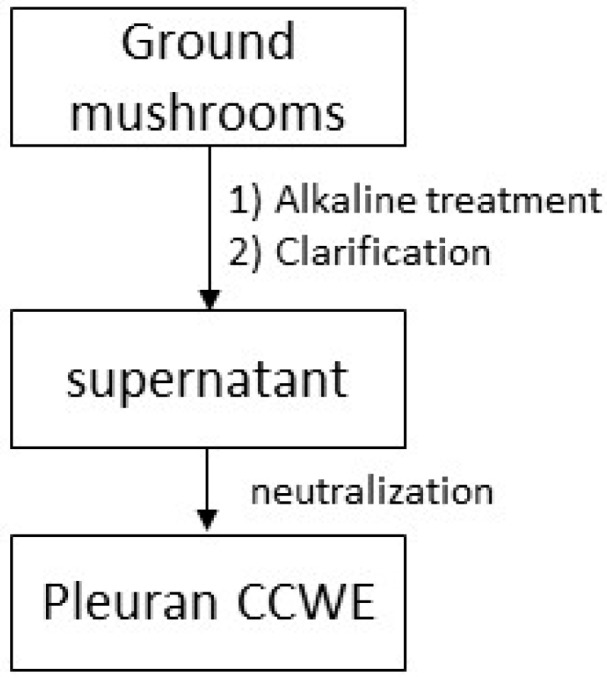
Pleuran CCWE extraction diagram.

Pleuran CCWE was obtained from dried *Pleurotus ostreatus* as described in Patent FR1874070 (see Section 5 and Figure 8).

### 3.3. β-Glucan Assay

The kit from Megazyme International (Bray, Co. Wicklow, Ireland), “MUSHROOM and YEAST B-GLUCAN” Assay Procedure K-YBGL 07/11 was used for glucans determination. The assay is based on the determination of total and α-glucans. β-glucans (non-starch) are determined by substraction. Total glucans are determined by quantification of glucose contents after the total acidic hydrolysis and α-glucans after specific enzymatic hydrolysis of starch-like α-glucans.

### 3.4. Crude Proteins

Total nitrogen was determined by the Kjeldahl method. The crude protein content was calculated by multiplying the N content by the factor 6.25 [25,26,27,28].

### 3.5. Monosaccharide Composition

15 µL of Pleuran CCWE extract were placed in a SVL tube and 100 µL of internal standard (myoinositol 200 µg/mL) were added. Tube contents were dried under N_2_ stream. Under an argon stream, 1 mL of MeOH/HCl 1 M was added and the stoppered tubes were heated at 80 °C during 24 h. After drying under a stream of argon, 100 µl of anhydrous pyridine and 100 µL of N,O-Bis(trimethylsilyl)trifluoroacetamide (BSTFA) were added, under argon atmosphere and the tubes were then heated à 30 °C during 2 h. Injections were performed in a Gas Chromatography with Flame-Ionization Detector (GC-FID)(Perichrom PR 2100, Perichrom, Saulx les Chartreux, France), equipped with a capillary column OPTIMA^®^-1-Accent 0.25 µM (Macherey-Nagel, Düren, Germany). Temperature of injector was 260 °C. Oven temperature program was 130 °C for 1 min, then increased to 190 °C (2 °C/min) for 5 min, then increased to 210 °C (2 °C/min) and finally increased to 260 °C (5 °C/min) and held during 10 min. Detector temperature was set at 260 °C.

### 3.6. SEC/MALS/DRI Analysis

Size-exclusion chromatography and multi-angle light scattering (SEC-MALS) (Wyatt Technology Inc., Santa Barbara, USA) analysis was realized as described by Picton et al. [29] and Simon et al. [30].

### 3.7. HPLC-SEC

Exclusion steric high-performance liquid chromatography (HPLC-SEC) analysis of Pleuran CCWE was carried out at room temperature by using a Chromoptic PL aquagel-OH (Chromoptic, Courtaboeuf, France) 20, 300 × 7.5 mm, 8 μm column, a Dionex P680 HPLC Pump (Dionex, Sunnyvale, CA, USA), a refractive index detector Shodex RI-101 (Showa Denko K.K., Tokyo, Japan), and a UV detector Dionex UVD170U (Dionex, Sunnyvale, CA, USA). Samples were eluted at a flow rate of 1 mL/min and 0.1 M NaNO3 as a mobile phase. Before HPLC analysis, Pleuran CCWE was incubated in 0.2 M NaOH during 90 min at 40 °C, as described by Peng and Zhang [31].

### 3.8. Plant Culture and Treatments

#### 3.8.1. Tomato Plants

Tomato seeds were sown in compost. Plantlets were grown in a greenhouse during one month and watered to maintain humidity. The plantlets harboring 2 expanded leaves, were treated with a solution of Pleuran CCWE (350 mg·L^−1^) by foliar spraying until run-off. Application was done three times at days 0, 2, and 4. On day 7, plantlets were inoculated with a conidial suspension of *Botrytis cinerea*, strain 117017 (UBOC), (10^5^–10^6^ spores per mL). Three spots of infiltration were performed on one of the first true leaves. Sample harvests were undertaken at different times depending on the analyzed parameter: on day 8 for gene expression quantification and on day 14 for peroxidase activities. Leaf samples were obtained from a leaf localized in the upper parts (and different from the inoculated leaf). They were immediately frozen in liquid nitrogen and stored either at −20 °C for enzymatic assays or at −80 °C for gene expression quantification.

#### 3.8.2. *Brachypodium Distachyon* Plants

*B. distachyon* ecotype Bd21 was used for this study. Plants were cultured as previously described by Pasquet et al. [32] for four weeks. Then, Pleuran CCWE extract solution was applied by spraying on spikelets three times at days 0, 2 and 5. *F. graminearum,* strain PH-1 (Fg DON+) was maintained on Potato Dextrose Agar (PDA) plates and a conidial suspension was obtained as described by Pasquet et al. [32]. Plant inoculation of was performed at day 7 (mid-anthesis stage, five weeks after sowing) by spraying all spikes with a spore suspension (10^5^ spores per mL).

### 3.9. Peroxidase Activity Quantification

Plant material was ground in a mortar in presence of 0.1 M sodium phosphate buffer pH = 7.0. After centrifugation (12,000× *g*, 10 min, 4 °C), the supernatant was assayed for peroxidase activity using 2,2′-azino-bis(3-ethylbenzothiazoline-6-sulfonic acid) (ABTS) as a substrate [33]. Kinetics were followed spectrophotometrically at λ = 412 nm. In order to determine the specific activity, the protein content of the supernatant was determined by the Bradford method [34].

### 3.10. Gene Expression

#### 3.10.1. Tomato Plantlets

Gene expression analysis in tomato plantlets was performed by Vegepolys Innovation (Angers, France) by using the quantitative RT-PCR microplate/DNA chip low density (QPFD^®^) method [35,36]. Briefly, after RNA extraction of leaves samples, extracts were spotted on FTA cards (Licence Nsure WO 2008018790, [37]). Reverse-transcription and quantitative real-time PCR were performed using the same patented set of primers for the 7 defense genes (*PR1, PR2, PR4,PR5, PR8, PR14* and *PR15*) and 3 reference genes (*GAPDH*, *actin* and *TuA*) [35]. Water control plants sampled the same day were analyzed and used as reference to determine the relative expression.

#### 3.10.2. *Brachypodium Distachyon* Plantlets

Two days post inoculation (day 9), a first set of ten plants were collected for RNA extraction and defense gene expression quantification. Reverse-transcription, and quantitative real-time PCR were performed as previously described by Gatti et al. [38]. Primers used to quantify expression of two defense genes: *PAL* and *PR9* (peroxidase) and two reference genes from *B. distachyon* ACT7 and UBC18 are presented in supplementary data 1. Two analytical replicates were considered.

At day 17 (10 days post-inoculation), symptoms were observed on a second set of ten plants. Spikelets were considered as symptomatic if 50% or more of floral cavities harbored fusarium symptoms. Also, plants were collected for analysis of fungal biomass. Quantification of fungal genomic DNA in infected spikes was performed as previously described by Pasquet et al. [32].

### 3.11. Field Trial

#### 3.11.1. Field Trial on Wheat

Field trial has been conducted on *Triticum aestivum* (Winter wheat, var. Rubisko) in Ecueillé (36240, France). Seed sowing was undertaken on 27 October 2017 on a total area of 500 m^2^, subdivided in 20 plots of 25 m^2^ (4 plots per treatment, randomly distributed).

Applications of Pleuran CCWE were performed by foliar spraying 3 times with the first application at Biologische Bundesanstalt, Bundessortenamt und Chemische Industrie (BBCH) 25 (T0 = 9 March), then at BBCH 31 (T1 = 30 March), and the last one at BBCH 37 (T2 = 29 April). Pleuran CCWE was prepared as a stock solution at a concentration of 35 g·L^−1^. Two doses were applied on wheat, either 1 L·ha^−1^, or 2 L·ha^−1^. Control plants (untreated) were sprayed with water at the same times as Pleuran CCWE (T0, T1 and T2). A treatment with two fungicides was performed and served as positive control. The first one was Cherokee^®^ (Syngenta, United Kingdom) containing 375 g·L^−1^ chlorothalonil, 62.5 g·L^−1^ propiconazole and 50 g·L^−1^ cyproconazole. The second one was Adexar^®^ (BASF, France) containing 62.5 g·L^−1^ epoxiconazole, 62.5 g·L^−1^ fluxapyroxad. Regarding the conventional program targeted against *Septoria tritici*, fungicides were positioned as usual i.e., Cherokee^®^ at BBCH 31 (T1) and Adexar^®^ at BBCH 37 (T2). Both were applied at a dose of 2 L·ha^−1^ (as recommended by manufacturers). All treatments (Pleuran CCWE or conventional fungicides) were undertaken in a final volume of 200 L·ha^−1^. No artificial contamination was attempted, thus Septoria development occurred naturally. Septoria symptoms were evaluated on 20 leaves per foliar level. Efficacy based on the percentage of attacked foliar area was calculated with Abbott’s formula (calculated on mean value): Efficacy % = 100 − ((Treated mean × 100)/Untreated mean).

#### 3.11.2. Field Trial on Grapevine

A field trial was conducted on grapevine (*Vitis vinifera*, var. Grenache noir) during the year 2015 in Villelongue-dels-Monts (66740, France) which had been planted at a density of 4000 vines per ha. Each elementary spot comprised 9 vine plants. For each treatment, 4 plots were randomly distributed among the field.

Grapevine were artificially contaminated with *Plasmopara viticola* on 17 May 2015 on the first vine plant of each individual plot. Also, aspersions were done during experiment to maintain humidity.

The applications of Pleuran CCWE were performed by foliar spraying six times between 12 May and 26 June, Table 4). Pleuran CCWE was prepared as a stock solution at a concentration of 35 g.L^−1^ and was applied by foliar spraying at a dose of 1 L·ha^−1^. To compare this treatment with a conventional fungicide, treatment with copper applied as Bordeaux mixture BB RsR Disperss NC (UPL Europe Ltd., Warrington, United Kingdom) containing 200 g Cu.kg^−1^ was also done and called hereafter Cu. According to the manufacturer’s recommendation, Cu was applied at a dose of 3.75 kg.ha^−1^ (or 750 g metal Cu per ha). Applications were done the same days as Pleuran CCWE (Table 4). In order to test the effects of a reduction of conventional fungicide, an alternative program was designed. It consisted in alternate applications of Pleuran CCWE and Cu. As this program led to a reduction of total Cu treatment, a partial check (called 1/2 Cu hereafter) has been done, consisting of three applications of Cu instead of six (Table 1). All treatments (Pleuran CCWE or conventional fungicides) were undertaken in a final volume of 200 L·ha^−1^.

On July 20th 2015, observations of symptoms of mildew attacks were done on grapes (50 grapes per elementary spot). Results were expressed in % of attacked grapes.

### 3.12. Statistical Analysis

Statistical analysis consisted of a one-way analysis of variance (ANOVA) test performed with the PAST software (version 2.17, University of Oslo, Oslo, Norway) to compare each set of plants with respect to the measured parameter. When significant difference was found (*p* < 0.05), Tukey’s test was carried out.

For field trial experiments, the statistical significances of differences among treatments were estimated using one-way ANOVA followed by Tukey’s test and the Student–Newman–Keuls test (*p* < 0.05).

## 4. Conclusions

An alkaline extract of the cultivated edible mushroom *P. ostreatus* has been obtained for the first time at laboratory scale. This extract is mainly composed of β-glucans (17.3%) and proteins or derivatives (23.4%). SEC-MALS and HPLC-SEC analyses confirmed that these polymers are closely associated in this extract, called Pleuran CCWE. The process has been successfully upgraded to pilot scale; the same extract was obtained with quite identical yield (about 30% of the dry raw material). Tested on plants by way of foliar spraying, Pleuran CCWE displayed remarkable elicitor activities which could be used to stimulate the plant defenses. Field trials confirmed the efficacy of Pleuran CCWE to limit the symptoms of pathogen-induced plant diseases as demonstrated on wheat and vine. Altogether, these results allowed us to propose Pleuran CCWE as an attractive alternative to pesticide use, in order to efficiently and safely protect cultivated plants against pathogen attacks in a more environment- and health-friendly way.

## 5. Patents

Célérier, J.; Moine, C.; Faugeron-Girard, C.; Gloaguen V. 2018. Substance biologiquement active, son procédé de fabrication et son utilisation comme agent protecteur d’un tissu biologique. Deposited on 21 December 2018 (FR 1874070).

## Figures and Tables

**Table 1:** Scaling-up from laboratory to pilot of Pleuran complex cell wall extract (CCWE) extraction process. Data are means +/− standard deviation (*n* = 23 for laboratory process; *n* = 12 for pilot process).

	Laboratory Process	Pilot Process
*Pleurotus ostreatus* (kg)	0.2	2.8
Volume produced (L)	1.7 +/− 0.2	22.3 +/− 1.6
Pleuran CCWE concentration (g/L)	32.9 +/− 5.0	40.7 +/− 2.9
Yield (% of dry raw material)	30.1 +/− 4.7	32.0 +/− 4.3

**Table 2:** Glucan and crude protein contents of Pleuran CCWE obtained with laboratory and pilot processes. Data are means +/- standard deviation (*n* = 23 for laboratory process; *n* = 12 for pilot process).

	% (*w*/*w* Dry Extract)	
	Laboratory	Pilot
β-glucans	15.5 +/− 4.1	17.3 +/− 4.5
α-glucans	1.4 +/− 1.0	1.5 +/− 0.8
Crude proteins (N × 6.25)	22.6 +/− 2.5	23.4 +/− 1.1

**Table 3:** Molar ratio (%) of monosaccharides in Pleuran CCWE. Data are means +/− standard deviation (*n* = 13 for laboratory process).

	**% Molar Ratio**	
	**Laboratory**	**Pilot**
Glucose	93.3 +/− 1.8	92.6
Mannose	3.9 +/− 0.8	4.1
Galactose	2.8 +/− 1.1	3.3

**Table 4:** Calendar of vine treatments during field trial. -: no treatment.

Dates	Development Stage^1^	Treatments				
		Control	Cu	Pleuran CCWE	1/2Cu + 1/2Pleuran CCWE	½ Cu
12 May	BBCH55	-	Cu	Pleuran CCWE	Cu	Cu
18 May	BBCH57	-	Cu	Pleuran CCWE	Pleuran CCWE	-
28 May	BBCH63	-	Cu	Pleuran CCWE	Cu	Cu
5 June	BBCH73	-	Cu	Pleuran CCWE	Pleuran CCWE	-
16 June	BBCH75	-	Cu	Pleuran CCWE	Pleuran CCWE	-
26 June	BBCH79	-	Cu	Pleuran CCWE	Cu	Cu

^1^ BBCH: Biologische Bundesanstalt, Bundessortenamt und Chemische Industrie.

## Supplementary Materials

The following are available online: Table S1: List of primers used in qRT-PCR experiments on *Brachypodium distachyon*.
