# Supplementary material for: Use of a Pleurotus ostreatus Complex Cell Wall Extract as Elicitor of Plant Defenses: From Greenhouse to Field Trial
Source: Molecules. 2020 Feb 29;25(5):1094. doi: 10.3390/molecules25051094 (PMC7179115; doi:10.3390/molecules25051094)
Supplement: Supplementary file 1 [file molecules-25-01094-s001.pdf]

Supplementary data

Table S1. List of primers used in qRT-PCR experiments on *Brachypodium distachyon*

Gene	Nucleotide sequence	Polarity	Putative function
Bradi4g00660	5'-ACCCTCTACGCTGGTGAGAC-3'	Forward	UBC18 (plant reference gene)
	5'-TTGCTGTAAATGTGCGGATG-3'	Reverse	
Bradi4g41850	5'-CCTGAAGTCCTTTTCCAGCC-3'	Forward	ACT7 (plant reference gene)
	5'-AGGGCAGTGATCTCCTTGCT-3'	Reverse	
Bradi1g39190	5'-TCCGACCAGGCTCTCTAC-3'	Forward	Pathogenesis-related protein PR9
	5'-GGTATGTTCCCCATCTTGAC-3'	Reverse	
Bradi3g47110	5'-CCAAACAATTAAGGAGATCAATTAGAA-3'	Forward	Phenylalanine ammonia-lyase
	5'- CCCGAATACTGGAAAGTAAGATACA-3'	Reverse	
